# A novel biplanar positioning technique to guide iliosacral screw insertion: a retrospective study

**DOI:** 10.1186/s12891-023-06482-y

**Published:** 2023-05-11

**Authors:** Yangyang Zhao, Pengju Cui, Zhenggang Xiong, Jiachun Zheng, Deguo Xing

**Affiliations:** Trauma Orthopedics, The Second Hospital of Shandong University, 247 Beiyuan Street, Tianqiao District, Jinan City, 250031 China

**Keywords:** Biplanar positioning technique, Iliosacral screw, Pelvic fracture, Ti-robot navigation

## Abstract

**Purpose:**

To evaluate the safety and benefits of the biplanar position technique on operative time, radiation exposure, and screw placement accuracy.

**Methods:**

In this study, we retrospectively evaluated the records of 64 patients with pelvic fractures (Tile B and C) between October 2020 and September 2021. According to the surgical methods selected by the patients, the patients were divided into a biplanar positioning technique group (biplanar group), a Ti-robot navigation group (Ti-robot group), and a traditional fluoroscopy-guided technique group (traditional group). Length of operation, blood loss, intra-operative radiation exposure fracture reduction, and the quality of screw positioning were compared among the three groups.

**Results:**

One hundred three screws were implanted in 64 patients (biplanar group 22, Ti-robot group 21, traditional group 21). The average operation time was significantly less in the biplanar group (26.32 ± 6.32 min) than in the traditional group (79.24 ± 11.31 min), but significantly more than in the Ti-robot group (15.81 ± 3.9 min). The radiation exposure was similar in the biplanar group (740.53 ± 185.91 cGy/cm2) and Ti-robot group (678.44 ± 127.16 cGy/cm2), both of which were significantly more than in the traditional group (2034.58 ± 494.54 cGy/cm2). The intra-operative blooding loss was similar in the biplanar group (12.76 ± 3.77 mL) and the Ti-robot group (11.92 ± 4.67 mL), both of which were significantly less than in the traditional group (29.7 ± 8.01 mL). The Screw perforation was slightly lower in the biplanar group (94.1%) than in the Ti-robot group (97.2%) but was significantly higher than in the traditional group (75.7%).

**Conclusions:**

The biplanar positioning technique is as accurate and safe as computer-navigated systems for percutaneous iliosacral screw insertion, associated with shorter surgical time, lower intra-operative radiation exposure, and more accuracy compared to traditional fluoroscopy.

## Introduction

Pelvic fractures represent 5—9.3% of all traumatic fractures, and unstable posterior pelvic ring injury incidence was 17%–30% [1–3]. As the posterior pelvic ring provides 60—70% of the stability of the pelvis, anatomical reduction with adequate fixation is crucial in cases with posterior pelvic ring injury [1, 4]. Percutaneous iliosacral(IS) screw fixation is widely used for posterior pelvic ring injuries and is becoming increasingly popular worldwide [5–7]. Despite percutaneous screw fixation has less surgical trauma, minimal blood loss, shorter operative time, fewer complications, and sufficient stability [8, 9], the traditional fluoroscopy-guided percutaneous technique has several concerns, including the damage from frequent X—rays, severe radiation exposure, high screw position error rate, and even nerve injuries [10].

In the past few years, several advanced real-time image tools, such as robot navigation systems, have been developed to improve the accuracy and safety of this technique [5, 11–13], but their high cost and complex technical equipment requirements in operation have limited their widespread application in intermediate and primary-care hospitals. The orthopedic surgical robot industry in China started relatively late. The market size in 2020 was $43 million, accounting for 10% of the overall market size of surgical robots [14]. Thus, these advanced equipment types may not be routinely available to each orthopedic surgeon and facility. Some surgeons have attempted to modify this traditional technique of IS screws to make it simpler and more feasible, such as three-dimensional-printed external templates, O-arm fluoroscopy, and intra-operative computed tomography [11, 15, 16].

**Fig. 1:**
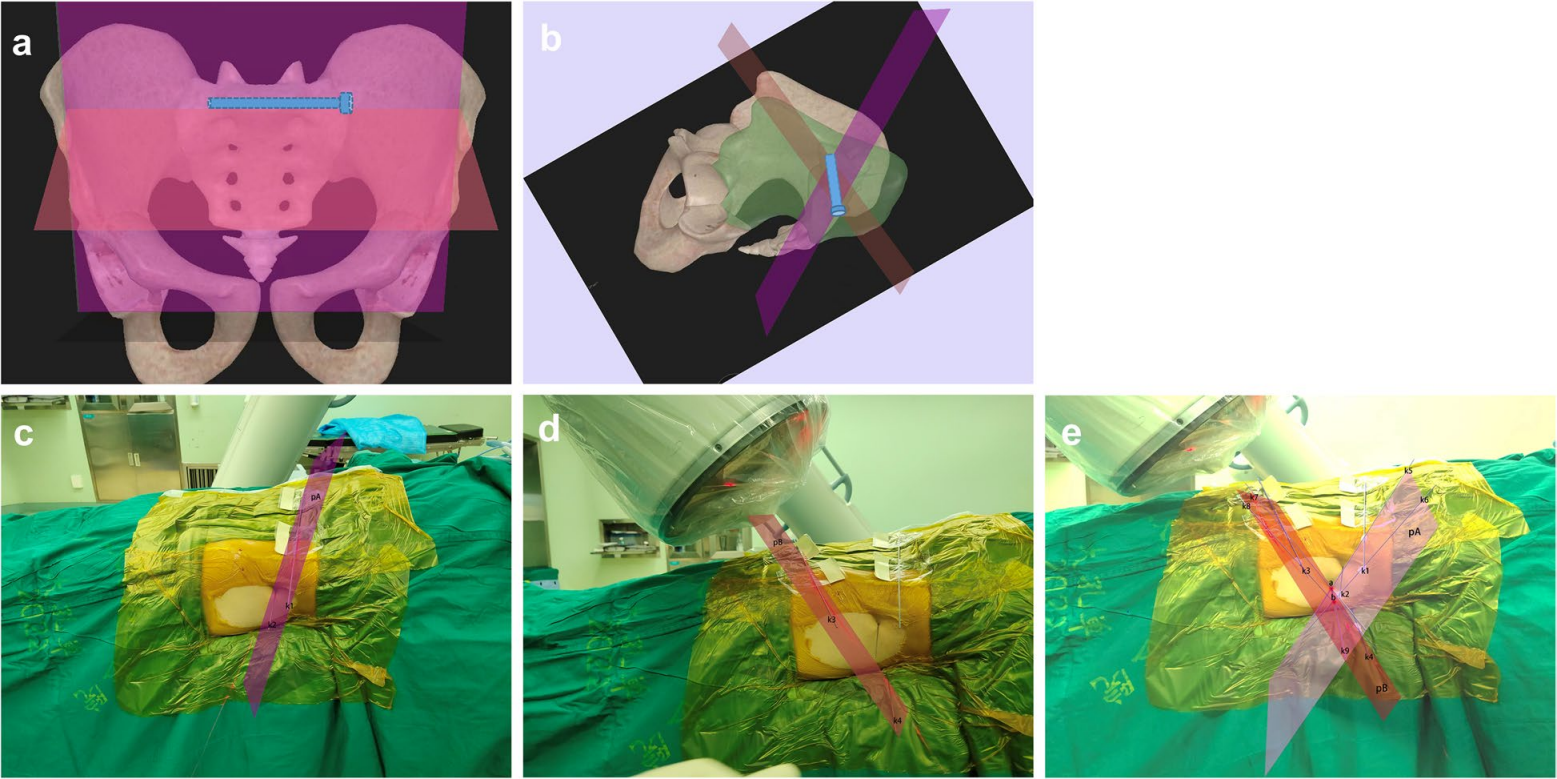
Schematic and demonstration drawing of the biplanar positioning technique. **a-b** The biplanar positioning is shown in the 3D reconstructed pelvis. **c-e** The biplanar positioning was simulated during surgery

Based on C-arm fluoroscopy, we first presented the application of the biplanar positioning technique in the placement of IS screws. The surgeon first obtained the satisfactory pelvic inlet and outlet plane during the surgery and marked them with two K-wires each. These intersecting K-wires defined the specific area of screw placement. The intersection line of the inlet and outlet planes was the locating line of IS screw. Then, this line was marked with a guide needle and an IS screw was placed along the guide needle (Fig. 1).

In this study, we retrospectively analyzed the safety and efficacy of the biplanar positioning technique in a series of patients with posterior pelvic ring injuries. The results were compared with traditional fluoroscopy and Ti-robot navigation techniques in terms of operation time, intra-operative radiation, screw insertion accuracy, etc. We thought the biplanar positioning technique would be safer and more maneuverable than the traditional fluoroscopy-guided technique.

## Methods

### Patients

Between October 2020 and September 2021, 64 consecutive patients with pelvic fractures (Tile B and C) were included in a cohort study at the Second Hospital of Shandong University. They were treated for traumatic incomplete or complete disruptions of the posterior pelvic ring using IS screws. IS screw placement, using the biplanar technique, was performed in 22 patients (biplanar group), including 13 men and nine women, 39.86 ± 16.07 years of age (range, 17–75 years). The Ti-robot-guide technique of IS screw insertion was used in 21 patients (Ti-robot group), including 11 men and ten women, 47.76 ± 17.03 years of age (range, 18–74 years). The traditional fluoroscopy-guided technique of IS screw insertion was used in 21 patients (traditional group), including 13 men and eight women, 48.05 ± 13.78 years of age (range, 22–73 years). All patients had experienced a high-energy trauma, with the distribution for all groups, respectively, as follows: high-energy fall, seven (31.8%, biplanar group) versus six (28.6%, Ti-robot group) versus eight (38.1%, traditional group); motor accident, 11 (50%, biplanar group) versus ten (47.6%, Ti-robot group) versus ten (47.6%, traditional group); crush injury, four (18.2%, biplanar group) versus five (23.8%, Ti-robot group) versus three (14.3%, traditional group). Using the Tile classification of pelvic fractures, the distribution of the type of posterior pelvic fractures for all groups, respectively, was as follows; Tile B fractures, 12 (54.5%, biplanar group) versus 13 (61.9%, Ti-robot group) versus 14 (66.7%, traditional group); Tile C fractures, ten (45.6%, biplanar group) versus eight (38.1%, Ti-robot group) versus seven (33.3%, traditional group). Demographic and clinical characteristics are shown in Table 1. After admission, the patient’s vital signs were monitored and intravenous access was established. Patients with hemodynamic instability were initially treated with temporary external pelvic fixation and admitted to the intensive care unit. In general, X-rays were performed 48 h after injury, when hemodynamic stability was achieved, to examine the effect of reduction, and surgery is performed on days three to ten.

**Table 1:** Demographic and surgery details

	Biplanar group (*n* = 22)	Ti-Robot group (*n* = 21)	Traditional group (*n* = 21)	*P* value
Sex (Male/Female)	13/9	11/10	13/8	0.813
Age (years)	39.86 ± 16.07	47.76 ± 17.03	48.05 ± 13.78	0.159
Tile classification				0.713
Type B	12	13	14	
Type C	10	8	7	
Injury mechanism				0.956
Fall	7	6	8	
Motor	11	10	10	
Crush	4	5	3	
Number of screws				0.535
S1	18	19	20	
S2	16	17	13	
Operation time (min)				< 0.001
S1	25.33 ± 6.66	15.74 ± 4.48	75.25 ± 11.53	
S2	27.44 ± 5.93	15.88 ± 3.26	85.38 ± 7.94	
Average	26.32 ± 6.32	15.81 ± 3.9	79.24 ± 11.31	
Radiation exposure (cGy/cm2)				< 0.001
S1	743.92 ± 209.34	684.92 ± 136.44	1933.14 ± 514.24	
S2	736.72 ± 162.31	676.74 ± 106.66	2190.65 ± 435.82	
Average	740.53 ± 185.91	681.06 ± 121.62	2034.58 ± 494.54	
Intra-operative bleeding (mL)				< 0.001
S1	12.22 ± 3.14	13.16 ± 4.5	28.8 ± 7.61	
S2	13.38 ± 4.4	10.53 ± 4.6	31.08 ± 8.71	
Average	12.76 ± 3.77	11.92 ± 4.67	29.7 ± 8.01	

### Biplanar positioning technique

**Fig. 2:**
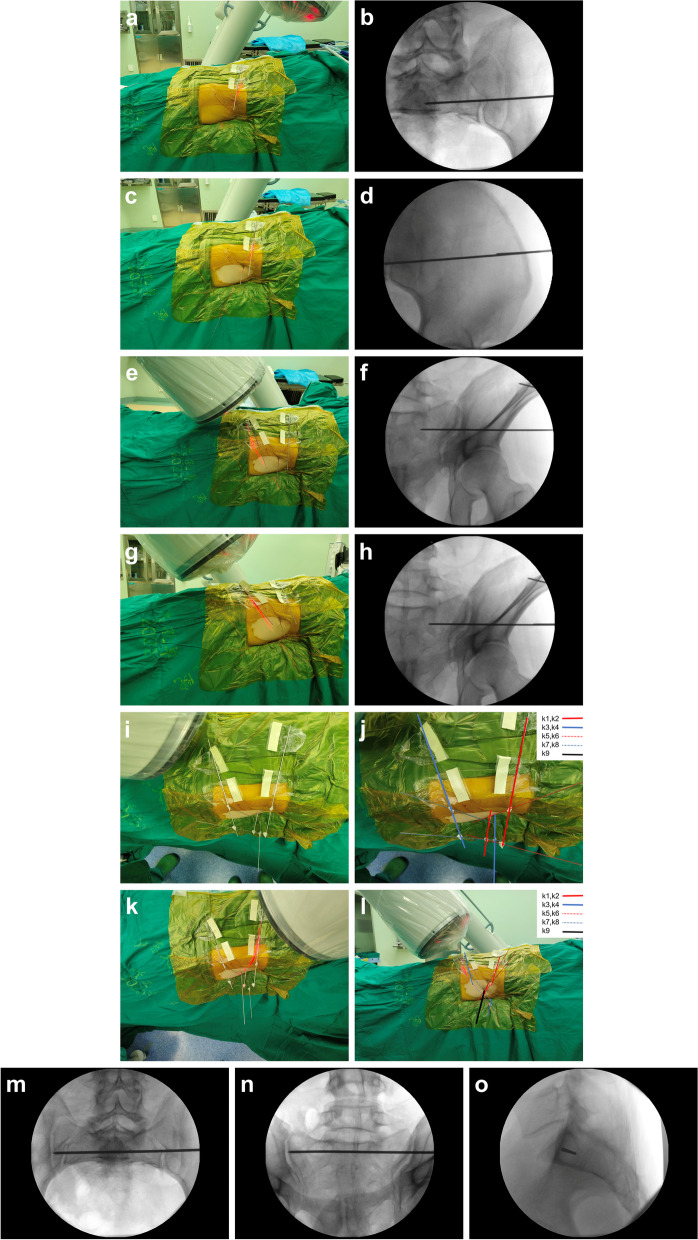
Surgical application of the biplanar positioning technique. **a**-**d** The k1 was secured with tape and the k2 was placed in the iliac crest so that the two K-wires coincided at the inlet view to determine the PA. **e**–**h** Tape k3 and k4 so that the two K-wires coincide at the outlet view to determine PB. **i**-**l** Line ab was determined by fixing k3, k4, k5, and k6 with bone wax. Line ab was the line (k9) where the IS screw is correctly positioned. **m**–**o** The position of the guide needle was confirmed by fluoroscopy

- ① The patient lies in the dorsal position on a carbon fiber operating table. C-arm fluoroscopy was placed contralateral to the surgeon. First, the inlet view of the pelvis was obtained through C-arm fluoroscopy. A K-wire (1.5 mm, k1) was placed on the patient's body surface and secured with tape, equivalent to the position and direction of the sacroiliac screw projected on the surface of the pelvic inlet. Another K-wire (1.5 mm, k2) was placed is placed within the patient’s ilium to make it coincides with k1 in the inlet view. According to the principle of two straight lines, a new plane was determined by k1 and k2, denoting PA. As in Fig. 2a-d, the k1 was secured with tape and the k2 was placed in the iliac crest so that the two K-wires coincided at the inlet view to determine the PA.
- ② With the same method, C-arm fluoroscopy was used to obtain a pelvic outlet view. A K-wire (1.5 mm, k3) was secured with tape, and placed on the body surface at the same position as the sacroiliac screw projected on the body surface in the outlet view. Place another K-wire (1.5 mm, k4) on the body surface so that it coincides with k3 in the outlet view. Thus, k3 and k4 determine a new plane, denoted as PB. As in Fig. 2e–h, tape k3 and k4 so that the two K-wires coincide at the outlet view to determine PB.
- ③ The intersection line of PA and PB planes is the position and direction of the sacroiliac screw placement. The other two K-wires (0.8 mm, k5, k6) were fixed on k1 and k2 with bone wax respectively so that k5 and k6 were in PA. In the same way, the other two K-wires (0.8 mm, k7, and k8) were fixed on k3 and k4 with bone wax respectively, so that k7 and k8 were in PB. Through the above operations, k5 and k7 intersect at one point (A), and k6 and k8 intersect at another point (B). The line between point A and point B (AB line) is the intersection line between PA and PB, and line ab is the correct location of the sacroiliac screw. As in Fig. 2i-l, line ab was determined by fixing k3, k4, k5, and k6 with bone wax. Line ab was the line(k9) where the IS screw is correctly positioned.
- ④ The guide needle (k9) slowly moves into the needle along line ab. At the same time, orient the guide needle in the inlet or outlet view for safety. The guide needle is gradually advanced until the optimum anatomical position is reached. After verifying the direction and position of the guide needle through the C-arm, an incision about 1-2 cm long was made at the needle entry point to separate the subcutaneous tissue and fascia muscle and reach the bone. After measuring the length, a cannulated drill bit was used to make the appropriate canal. The IS screw was then inserted along the guiding needles. The position of the screws was confirmed through C-arm fluoroscopy again. As in Fig. 2m–o, the position of the guide needle was confirmed by fluoroscopy.

### Ti-robot guided technique

The patient lies in the dorsal position. according to the Ti-robot system operation process, inlet, outlet, and lateral views were captured for positioning, and the sacroiliac screw placement path was planned according to the patient's anatomical features and fracture status. the robotic arm moves the guiding sleeve to the planned area. The surgeon made a 1.5 cm incision and inserted the drill sleeve until the tip was pushed tightly on the bone surface. A guiding needle was inserted through the drill sleeve, and the needle path was verified through fluoroscopy. Subsequently, a cannulated drill was used to expand the path. Through the guiding needle, an IS screw was screwed in. The position of the screws was again confirmed through fluoroscopy.

### Traditional fluoroscopy technique

The patient lies in the dorsal position. According to the C-arm standard lateral fluoroscopy, the guide needle was drilled horizontally into the external iliac bone plate at the midpoint of the lateral view of S1. The direction of the guide needle was evaluated under the inlet view, and then its horizontal direction in the sacrum was estimated under the outlet view. The needle was slowly advanced and fluoroscopy was performed continuously to confirm the guide needle until the optimal anatomical location was reached. After estimating the length, a cannulated drill bit was used to expand the path. The IS screw was then screwed in. The position of the screws was again confirmed through C-arm fluoroscopy.

### Post-operative treatments

The post-operative regimens were similar between the three groups. All patients were provided symptomatic treatment, infection prevention, and electrolyte balance. According to the actual situation of the patients, early functional exercises were performed under the guidance of physical therapists. 6—8 days after the operation, the patients were allowed to turn in bed with the assistance of families. Meanwhile, the patients gradually practiced active contraction of muscles in both the lower limbs and hip and knee flexion.

### Measurement

**Fig. 3:**
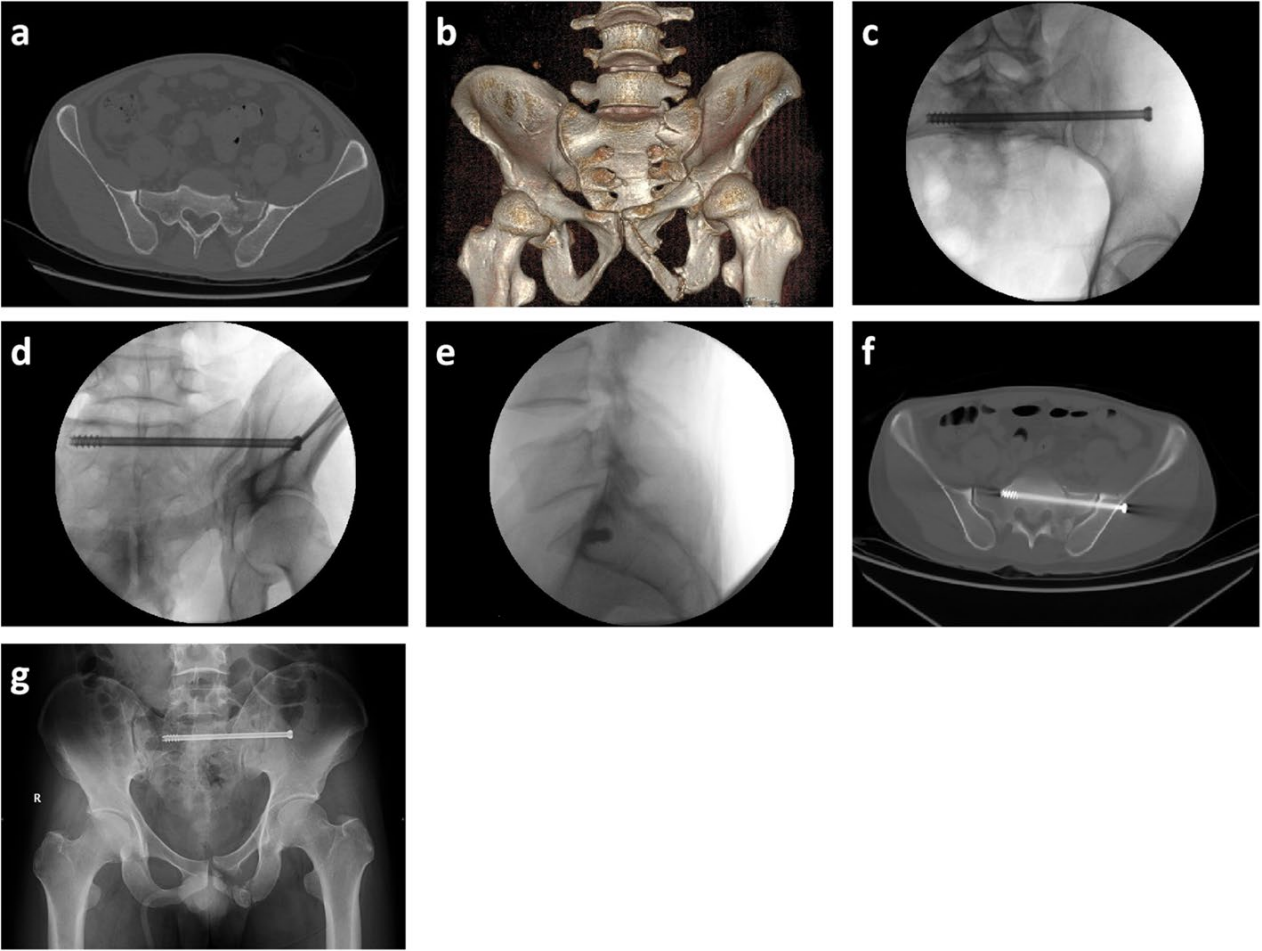
Pre-operative, intra-operative, and post-operative imaging. **a-b** Pre-operative computed tomography images/ 3D reconstruction for a patient with Tile B2.1 pelvic fractures. **c-e** Intra-operative imaging (inlet/outlet/lateral view) was used to confirm the IS screw in the target corridor. **f-g** Post-operative computed tomography axial image and anterior–posterior radiograph computed tomography axial image confirmed the placement of the IS screw

A review was undertaken of the pelvic radiographs and CT images (Fig. 3). All the screws were measured by each observer who did not participate in the operation. According to the following grading criteria [17], the final screw position from post-operative CT images was estimated by consensus among the three observers:

1. grade 0, no violation;
2. grade 1, < 2 mm;
3. grade 2, 2–4 mm;
4. grade 3, > 4 mm.

The quality of the reduction was evaluated according to the maximum displacement degree at the CT images, using the following Matta grading criteria [18], as previously described:

1. Excellent, ≤ 4 mm;
2. Good, 4–10 mm;
3. Fair, 10–20 mm.

The amount of time required for screw insertion and radiation exposure dose were also extracted from the surgical records for analysis.

### Statistical analysis

The SPSS version 25.0 software program was used for the statistical analyses. Quantitative data are presented as the mean ± standard deviation. Between-group differences were evaluated using AVONA, the chi-squared test, and the Kruskal–Wallis tests, as appropriate for the data type and distribution. In all analyses, a value of p < 0.05 was accepted as statistically significant.

## Results

### General results

A total of 103 screws were inserted in 64 patients, with an average of 1.6 screws per patient: 34 screws (18 S1, 16 S2) in the biplanar group (22 patients), 36 screws (19 S1, 17 S2) in the Ti-robot group (21 patients), and 33screws (20 S1, 13 S2) in the traditional group (21 patients). There was no statistical difference in the quality of reduction among the three groups (Table 2).

**Table 2:** Quality of the reduction

	Excellent (≤ 4 mm)	Good (4-10 mm)	Fair (10-20 mm)	*P* value
Biplanar group	5	14	3	0.724
Ti-Robot group	6	13	2	
Traditional group	4	14	3	

### Operation time

The average operation time per screw was 26.32 ± 6.32 min (range, 18-43 min) in the biplanar group and 15.81 ± 3.9 min (range, 10-26 min) in the Ti-robot group, both of which were significantly less than the 79.24 ± 11.31 min (range, 55-95 min) required in the traditional group (*p* < 0.001).

### Radiation exposure

The radiation exposure dose per screw was 740.53 ± 185.91 cGy/cm^2^ (range, 471.5–1225.9 cGy/cm^2^) in the biplanar group and 678.44 ± 127.16 cGy/cm^2^ (range, 377.2–943.0 cGy/cm^2^) in the Ti-robot group, both of which were significantly less than the2034.58 ± 494.54 cGy/cm^2^ (range, 1320.2–2829.0 cGy/cm^2^) in the traditional group (*p* < 0.001). There was no statistical difference in radiation exposure between the biplanar group and the Ti-robot group (*p* > 0.05).

### Intra-operative blooding loss

The intra-operative blooding loss per screw was 12.76 ± 3.77 mL (range, 5–21 mL) in the biplanar group and 11.92 ± 4.67 mL (range, 5–22 mL) in the Ti-robot group, both of which were significantly less than the 29.7 ± 8.01 mL (range,18–45 mL) in the traditional group (*p* < 0.001). There was no statistical difference in this blooding loss data between the biplanar group and the Ti-robot group (*p* > 0.05).

### Screw perforation

The rate of screw perforation was also lower in the biplanar (two of 34 screws, two at grade 1) and the Ti-robot (one of 36 screws, one at grade 1) than the traditional (eight of 33 screws, five at grade 1, three at grade 2) group (*P* < 0.001, Table 3). There was no statistical difference in screw perforation between the biplanar and the Ti-robot groups (*p* > 0.05). No incidence of neurovascular injury occurred among cases of screw perforation. No incidence of loosening and fractures of screws was found among all patients.

**Table 3:** The rate of screw perforation

	Grade 0	Grade 1	Grade 2	*P* value
Biplanar group	94.1%	0.59%	0%	0.008
S1	17	1	0	
S2	15	1	0	
Ti-Robot group	97.2%	2.8%	0%	
S1	19	0	0	
S2	16	1	0	
Traditional group	75.7%	15.2%	9.1%	
S1	16	3	1	
S2	9	2	2	

## Discussion

As confirmed by our study, the use of the biplanar positioning technique to insert IS screw provided high accuracy, with a shorter operative time, less blood loss, and lower radiation exposure than the traditional fluoroscopy-guided technique. Compared to the Ti-robot group, surgical time for the biplanar positioning technique was significantly prolonged at about 11 min; however, this time is acceptable for a new technique.

During the operation, two different views should be used to evaluate the anterior and posterior borders of the sacral body. However, any adjustment in the position of the guide wire required in one view should necessarily be reconfirmed on all fluoroscopic views [19–21]. In the previous study, Rane et al. [22] described that attempting to change the position of the guide wire perpendicular to the fluoroscopy beam in either view would lead to unintentional biplanar motion in the other view. Correcting these unintentional biplanar movements requires repeatedly switching between the inlet and outlet views after each positional change. Unfortunately, this procedure greatly increases operative time and radiation exposure. However, this unintended biplanar motion can be avoided by moving the guide needle parallel to the angle of either the inlet view or outlet view [22]. Inspired by the above content, we innovatively put forward the simple biplanar positioning technique. Only four K-wires and a few fluoroscopies are needed to get the correct inlet and outlet planes. Since the intersection of two planes determines a straight line, the intersection line of the inlet and outlet planes was the locating line of IS screw. Then, we can use K-wires to position this line outside the body without fluoroscopy.

The operation time in the biplanar group was significantly lower than those in the traditional group. In this study, although the operation time was significantly about 11 min longer in the biplanar group than in the Ti-robot group, the operation time of the Ti-robot group did not include the time to assemble the robot. In the study by Zwingmann et al. [23, 24], screw placement time was compared between navigational surgery and traditional screw placement surgery, and there was no statistically significant difference between the two groups. Based on our retrospective analysis, we found no data recorded on setup times in our records. Therefore, we cannot compare this parameter. Generally, it takes approximately ten extra minutes to install the navigation system in the operating room, and extra 7.8 min to plan screw trajectories [23, 25].

Radiation exposure is a significant concern for percutaneous IS screw insertion. Excessive amounts of radiation are detrimental to surgeons, patients, and operating room staff [26, 27]. After positioning the inserting line by the biplanar positioning technique, fluoroscopy can be avoided during the inserting process. If necessary, the input and lateral view can verify the screw position and direction. The main aim of biplanar positioning technology is to reduce intra-operative radiation exposure, which is especially important for the surgeon exposed regularly.

In general, the accuracy and safety of IS screw insertion have been evaluated using the penetration grades [17]. The penetration grade was not significantly lower for the biplanar group than the Ti-robot group, but it was significantly higher than the traditional group. At present, the accuracy and malposition rate of IS screw insertion between the traditional method and Ti-robot did not reach a consensus. Because of the wide range of malposition rates [28], the variation is mainly related to differences in the definition of malposition. A screw is frequently defined as “malpositioned” when the screws inserted are insecure and the patient needs revision surgery. Some studies showed the opposite results [29, 30]. Most studies reported the malpositioning rate is lower under the computer-navigated technique [29, 31]. However, in a meta-analysis reported by Zwingmann et al. [32], no significance in the revision rate was observed between the traditional (2.7%) and computer-navigated technique (1.3%). Berger-Groch et al. [29] reported in a retrospective study the same malpositioning rate on 232 screws for traditional and computer-navigated iliosacral screw placement. We believe the penetration grades may have a ceiling effect. If the presence of most screws does not penetrate the cortical bone, this classification may reduce its accuracy. Takao et al. [33] described a method to measure the deviation distances of planned and actual screw tips, nerve root tunnel areas, and entry points on sagittal CT images. They reported mean deviations of 2.2 ± 0.8 mm (tip), 1.8 ± 0.7 mm (nerve root tunnel), and 2.5 ± 1.8 mm (entry point). However, We believe that this method is only helpful for the accuracy of screw placement, and has no obvious effect on the evaluation of screw placement in safety.

As an outstanding product in the intelligent era, now, robot navigation technology is popular in general hospitals, with its safe and minimally invasive. Due to the high cost and complex technical equipment requirements in operation, robot navigation technology was limited to their widespread application in intermediate and primary-care hospitals. The orthopedic robot system cost $210,800–281,000, or even more than $800,000, and an orthopedic robot operation requires an additional $1000 for equipment usage (start-up charge). The annual maintenance cost is about $21,000 or more [34, 35]. There are costs associated with training technicians. These costs may vary geographically but must be considered in the overall cost. However, the biplanar positioning technique has a relatively low threshold, simple operation, and fewer equipment requirements. And any hospital equipped with C-arm fluoroscopy can perform the procedure, especially in less developed areas. In addition, poor patients with pelvic fractures can afford this minimally invasive surgery.

Due to the irregular shape of the pelvis, the surgeons are unable to see the internal structure and obtain real-time 3D images of the surgical site during the surgery, so the traditional technique of IS screw requires the surgeon's 3D spatial imagination ability, and there is a high risk of injury the surrounding tissue. For the new robot navigation technology, clinicians must need strict and formal training before they carry out this technology. They must strictly master the surgical indications and operate by the standard operation process to ensure the accuracy and safe of the navigation system and the smooth progress of subsequent surgical operations. And the operator's proficiency and the operation team's tacit understanding are also very important. The above result, there is a long learning curve for this technique [34]. On the contrary, the biplanar positioning technique can split the positioning of IS screws from three-dimensional space to two planes, which greatly reduces the difficulty, so the technique is easier for doctors to master. This technology has a short learning curve, and we found that when surgeons mastered this technique, they became more adept in traditionally placing the IS screws. The biplanar positioning technique has been applied to other fractures, including acetabular fractures involving anterior and posterior columns, femoral neck fractures, scaphoid fractures, etc. At present, the biplanar positioning technique is still composed of multiple K-wires, which do have some instability. However, its stability can meet the requirements of the surgery. On this basis, we are developing an integrated frame with biplanar positioning technology, which is expected to greatly improve its stability.

In conclusion, percutaneous IS screw placement by biplanar technique to treat posterior pelvic ring fractures is safe, convenient, and accurate. In addition, the biplanar technique is a safe and effective method that shortened the operative time and reduced radiation exposure. Our suggested technique will hopefully help surgeons improve the efficiency and accuracy of IS screw placement. This technique will also hopefully help this minimally invasive surgery to be available in basic hospitals that are not equipped for robotic navigation and be affordable for poor patients.

## Limitations

There are some limitations to this study. Because of the retrospective study design, the patients were not randomly allocated to each group. Although systematic bias could not be avoided, the random allocation would certainly have increased the accuracy of the conclusions made. The bi-planar technique is a new technology in our institute and the number of cases included in the study was small. The above results are for early work, the follow-up time was relatively short, and further experience needs to be gained in more clinical cases.

## Data Availability

The datasets used and/or analyzed during the current study are available from the corresponding author on reasonable request.
